# Multiple organism algorithm for finding ultraconserved elements

**DOI:** 10.1186/1471-2105-9-15

**Published:** 2008-01-11

**Authors:** Scott Christley, Neil F Lobo, Greg Madey

**Affiliations:** 1 Department of Computer Science and Engineering, University of Notre Dame, Notre Dame, IN, 46556, USA; 2 Interdisciplinary Center for the Study of Biocomplexity, University of Notre Dame, USA; 3 Department of Biological Sciences, University of Notre Dame, Notre Dame, IN, 46556, USA

## Abstract

**Background:**

Ultraconserved elements are nucleotide or protein sequences with 100% identity (no mismatches, insertions, or deletions) in the same organism or between two or more organisms. Studies indicate that these conserved regions are associated with micro RNAs, mRNA processing, development and transcription regulation. The identification and characterization of these elements among genomes is necessary for the further understanding of their functionality.

**Results:**

We describe an algorithm and provide freely available software which can find all of the ultraconserved sequences between genomes of multiple organisms. Our algorithm takes a combinatorial approach that finds all sequences without requiring the genomes to be aligned. The algorithm is significantly faster than BLAST and is designed to handle very large genomes efficiently. We ran our algorithm on several large comparative analyses to evaluate its effectiveness; one compared 17 vertebrate genomes where we find 123 ultraconserved elements longer than 40 bps shared by all of the organisms, and another compared the human body louse, *Pediculus humanus humanus*, against itself and select insects to find thousands of non-coding, potentially functional sequences.

**Conclusion:**

Whole genome comparative analysis for multiple organisms is both feasible and desirable in our search for biological knowledge. We argue that bioinformatic programs should be forward thinking by assuming analysis on multiple (and possibly large) genomes in the design and implementation of algorithms. Our algorithm shows how a compromise design with a trade-off of disk space versus memory space allows for efficient computation while only requiring modest computer resources, and at the same time providing benefits not available with other software.

## Background

The availability of whole genome assemblies [1-3] and the development of bioinformatics tools and interfaces [4,5] for their analysis, enable data-mining and comparison of these large genomic datasets. Ultraconserved elements are nucleotide or protein sequences with 100% identity (no mismatches, insertions, or deletions) in the same organism or between two or more organisms. A recent comparison of several vertebrate genomes demonstrates that, in addition to coding, non-coding sequences can be highly conserved between species [6]. Approximately 5% of the human genome is under negative selection, indicating conservation of sequence due to functional necessity. These functional regions are conserved since random mutations that would negatively effect functionality would be rejected by natural selection. Consequently, orthologous functional regions would be more similar between different genomes. Genome comparison has become a vital tool in the identification of these conserved functional elements. Of the 5% of the human genome that is under selection, only 1.2% codes for protein sequences [6]. Surprisingly, the most conserved regions amongst vertebrates are in non-coding sequences. These ultraconserved elements are significantly more conserved (up to 100%) than would be expected across genomes. Though these ultraconserved elements seem to be specific to vertebrates, they are present in other metazoan genomes as well [7-9]. Studies indicate that these conserved regions are associated with micro RNAs, mRNA processing, development and transcription regulation [7-13]. The identification and characterization of these elements among genomes is necessary for the further understanding of their functionality. Multiple organism whole genome comparative analyses such as these would elucidate the fun! ction of these conserved elements and their importance in various evolutionary lineages.

In this paper, we describe an algorithm, a set of freely available software programs, and a workflow for finding ultraconserved elements for multiple organisms. We explicitly design our algorithm to take both computational time and memory space in to account such that it can handle genomes of any size as well as take advantage of parallel computation on typical computer clusters. Our algorithm and workflow provides a case study showing that by focusing on a specialized task, computation can be orders of magnitude more efficient; this allows previously impractical, but highly desirable, whole genome comparative analysis to be performed.

## Results and Discussion

Algorithms that perform multiple organism comparative genomics must explicitly deal with the issue of large genomes. Most vertebrate genomes, such as human, mouse, chicken, etc., approach the maximum size of addressable memory space for 32-bit processors (4 GB). 64-bit processors which have sufficiently large addressable memory space (millions of GB) generally do not have more than 16–32 GB of actual physical memory due to technology limitations or because the cost is prohibitive. This physical memory bound will increase in the future as memory capacities increase, but for the near future, the generation of genomic data is increasing at an even faster rate. Even if all the genomic data can be brought into memory, there still needs to be space for data structures that organize the data for algorithmic purposes; these data structures are often multiple times larger than the raw genome data.

### Algorithm

**Figure 1:**
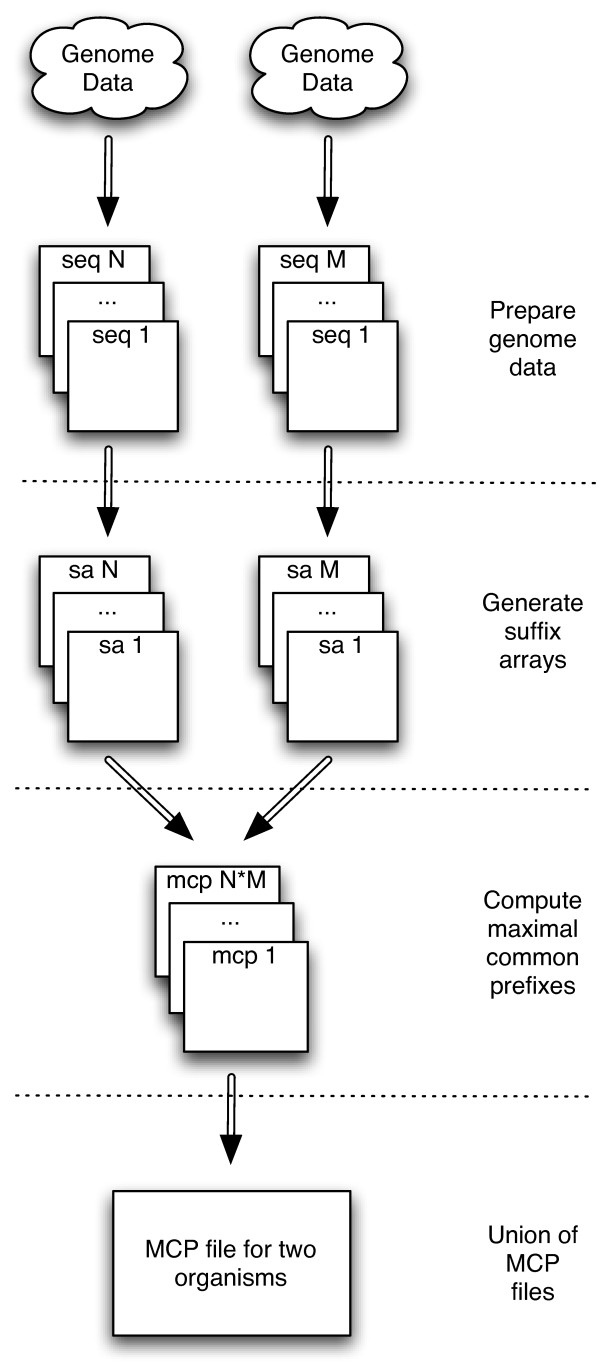
**Workflow for Computing Maximal Common Prefixes between Two Organisms**. The steps for computing the maximal common prefixes between two organisms involves preparing the raw genome data into a set of sequence files in FASTA format, generating suffix array data structures from the sequence files, computing the MCP's in a pairwise fashion with the suffix arrays, then perform the union of the pairwise files together to produce a single MCP result file.

**Figure 2:**
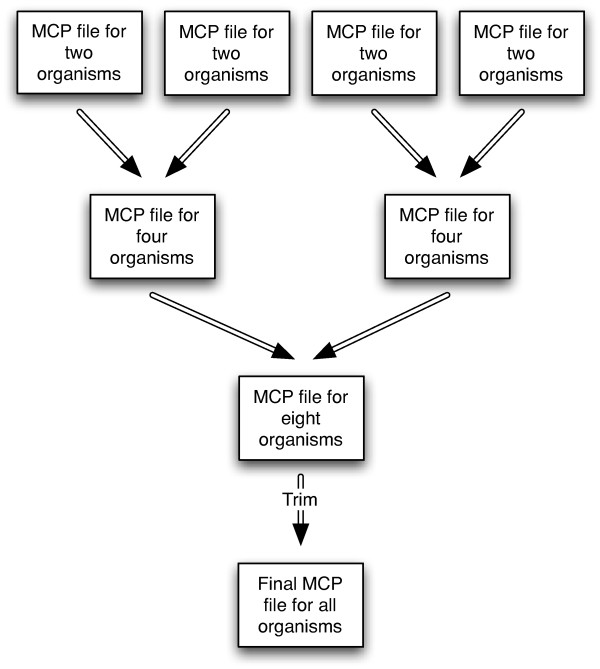
**Tournament-style Intersection for Computing Maximal Common Prefixes between Multiple Organisms**. A generic workflow for intersecting the MCP's in a tournament-style to produce the common sequences for any number of organisms. Typically the number of MCPs becomes less than the total number of suffixes as more organisms are intersected so the later stages of the workflow execute faster than the earlier stages. This generic workflow can be easily modified to support more automated specialized processes, for example comparing all the organisms within the clade of a phylogenetic tree, because the output of each stage can be directly input to the next stage without any additional processing. Trimming the file is a separate step only needed for reporting final results and does not alter the MCP file allowing it to be used for continual stages of the workflow.

Our algorithm for finding the longest ultraconserved sequences for multiple organisms breaks up the task into smaller, manageable subtasks which in most cases can all be run in parallel on a computer cluster. The first step is to prepare the genome data into appropriate size data files. If the genome has already been mapped to chromosomes then the individual chromosome sequences provide a natural division; otherwise, the genome is probably provided as one large FASTA file containing all of the assembled scaffolds. The next step is to generate a suffix array data structure for each data file. The main algorithm then takes two suffix arrays and produces a list of maximal common prefixes (MCP) which correspond to ultraconserved sequences; this is done in a pair-wise fashion for all suffix arrays of the two organisms. The union of the MCP files produces the final MCP file for the two organisms. The workflow is summarized in Figure 1. For multiple organisms, each final MCP file for a pair of organisms is intersected within another final MCP file for a different pair of organisms; this is repeated in tournament-style fashion producing an MCP file for all the organisms. Lastly the MCP file can be trimmed to remove overlaps and retain just the longest sequences. Figure 2 shows the tournament-style intersection for multiple organisms. We describe each step in detail in the following sections.

#### Prepare Genome Data

The genome data for each organism can be split into appropriate size sequence files to facilitate parallel execution of many of the programs in the workflow. If the genome has already been mapped to chromosomes then the individual chromosome sequences provide a natural division. Otherwise, the genome is probably provided as one large FASTA file containing all of the assembled scaffolds; in which case, that single file may be split into several smaller files. We provide a simple utility, **split_fasta**, which will split up a FASTA file containing numerous sequences into several files. The largest memory requirement is incurred when the genome data is processed into suffix array data structures. The following calculation can be used to determine how much memory will be required for each strand:

*Memory*(*bytes*) = *Sequence_size *+ (2 * Sequence_size * *sizeof*(*int*))

where *Sequence_size *is the number of nucleotide base pairs in the sequence. The size of the integer data type, (*sizeof*(*int*)), on most platforms is 4 bytes. The first term is the size of the sequence, and the second term is the size of the suffix array which is doubled for temporary storage required by the sorting process. For example, human chromosome 1 is ~250 MB which gives the memory requirement:

*Memory *= 250 *Mbp *+ (2 * 250 Mbp * 4) = 2.25 *GB*

When constructing the suffix array from the sequences (described in the next section), any size sequence file can be accommodated regardless of the available physical memory up to the virtual memory limit of the machine. However if the memory usage as required by the above equation exceeds physical memory then construction of the suffix array will take longer, so to maximize efficiency the size of the sequence files can be reduced so that the processing of each individual file fits completely into the physical memory of the machine. This memory usage is only for the initial creation of the suffix array; afterwards the suffix array is stored on disk and memory requirements are minimal for the additional processing steps of the workflow. There is no limit to the number of individual files that can be processed with the algorithm, so it is better to have many smaller files than just a few large files.

#### Generate Suffix Array Data Structures from Genome Data

**Figure 3:**
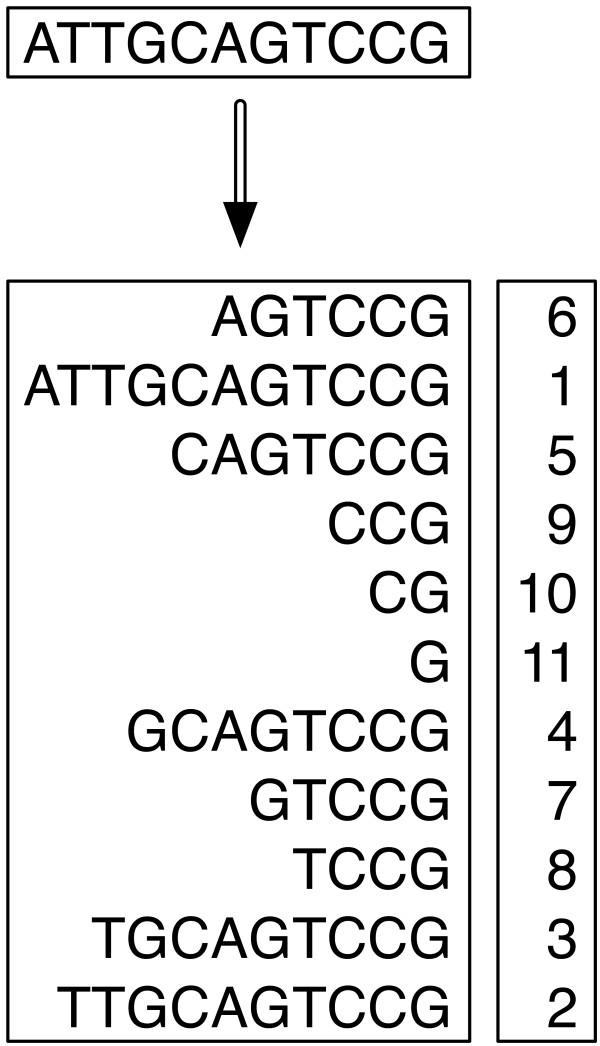
**Suffix Array from Sequence**. A sequence of length N produces N suffix strings which are sorted in the suffix array data structure as shown with this 11 bp sequence and its corresponding suffix array. Each suffix string does not have to be stored separately, instead character positions or offsets are used to reference back to the original sequence; the offsets are the numbers to the right of each suffix.

We process each sequence file into a specialized data structure called a suffix array. A suffix array [14] looks at the sequence as one long string, takes all possible suffix strings, and sorts those suffix strings in lexicographical order. A suffix is a trailing end substring, so a string of length N has N unique suffix strings of length N, N-1, N-2, etc. We use an implementation for computing a *suffx *array by Sean Quinlan and Sean Dorward [15] that can sort all of the suffix strings in-place using offsets into the full string thus making for efficient sorting, though the whole suffix array does need to fit into memory. If the sequences and resulting suffix array are too large for the available physical memory, then multiple suffix arrays are created on partitions of the sequence data, and those multiple arrays are merged together to form a single result file. The resultant suffix array is a list of offsets into the original sequence string. Figure 3 shows an example string with its corresponding suffix array. We use a suffix array instead of a suffix tree because it is more amenable for storage and access in a file; thus we do not require the whole data structure to be present in memory.

We provide two programs for constructing the suffix array for a sequence, **construct_sarray **and **construct_prot_sarray**. The first program takes a nucleotide sequence, concatenates the sequence for the reverse strand, computes the suffix array, and then writes the suffix array along with some reference data to files. The other program takes a nucleotide sequence, translates codons according to a user-specified genetic code translation table into amino acids for the three reading frames on the forward strand and the three reading frames on the reverse strand, computes the suffix array, and then writes the suffix array along with some reference data to files. Both programs take into account repeat masked sections of the sequence, whether masked with N's or lower case nucleotide letters, by removing suffix strings that start with repeat masked nucleotides or by not translating those sections.

Each individual sequence file is processed into its own suffix array, so the human genome with 22 chromosomes plus the X and Y chromosomes would be processed into 24 separate suffix array files. If the human genome was also translated then there would be another 24 suffix array files for the translated nucleotide sequences. The primary tradeoff is between the use of disk space in exchange for lower memory requirements, but with appropriate data structures the file-based computation is very efficient. There is no dependence between suffix arrays for different sequence files, so all of the suffix arrays can be computed in parallel on a typical computer cluster.

#### Compute Maximal Common Prefixes between Two Organisms

The maximal common prefix (MCP) for two lists of strings is the problem of finding a string in one list that has a common prefix with a string in the other list, and that common prefix is the maximum length for all possible choices of strings. A naive implementation would require each string in one list to be compared to all strings in the other list resulting in quadratic *O*(*n*^2^) running time. However with our strings in sorted order in a suffix array, we can compute the maximal common prefixes with just a linear scan through both suffix arrays. Such an algorithm can find not only the single MCP but all MCP's for all strings.

We provide the program, **mcp_sarray**, which given two suffix array files will output all maximal common prefixes greater than or equal to a user-specified length. These maximal common prefixes correspond to ultraconserved sequences as each MCP is an equivalent sequence that appears in both organisms. The program works for both nucleotide and protein sequences. In the first step, the organism's genome was separated into appropriate size sequence files, so we have multiple suffix array files for the organism. Finding all of the MCP's requires running the program in a pairwise fashion with all suffix array files for both organisms. For example, the human genome has 24 suffix array files and the mouse genome has 21 suffix array files, so pairwise application will generate 504 resultant MCP files. The program, **union_mcp**, will combine together these individual files into a single resultant MCP file. One may consider combining all of the suffix array files for an organism into a single suffix array so that the **mcp_sarray **is just run once; however there are considerably fewer MCP's making the MCP files much smaller than their corresponding suffix array files, so it is more efficient to combine results afterwards rather than before. Furthermore, there are no dependencies between the pairwise **mcp_sarray **programs, so they can be executed in parallel on a computer cluster which is faster than just two large suffix array files. The pairwise MCP files can be considered temporary files and deleted once they are combined together into a single file. However, care should be taken when running many programs in parallel as disk I/O can quickly become a bottleneck if the suffix array files are stored on shared disk space. Efficiency can be increased by copying the suffix array files to local disk on each compute node, and the **mcp_sarray **program attempts to bring in sequence data into memory as a tradeoff between memory and disk I/O.

#### Compute Maximal Common Prefixes for Multiple Organisms

Computing the MCP's for multiple organisms involves taking the MCP file for two organisms and intersecting it with the MCP file for two other organisms, thus producing the maximal prefixes common to all four organisms. This can be repeated in a tournament-style fashion for any number of organisms. Similar to the algorithm for computing the MCP's between two organisms, the intersection algorithm for multiple organisms can be performed with just a linear scan through both MCP files. We provide the program, **intersect_mcp**, which given two MCP files will output all maximal common prefixes that are present in both input files.

The format of the MCP file is similar to a suffix array but requires additional data to support any number of organisms. This additional data includes references to a particular sequence for each organism, an offset into each of those sequences, and a length corresponding to the current maximal common prefix. The sequences in the MCP file are maintained in sorted order. Intersection of two MCP files proceeds with a linear scan through both files, determining the maximal common prefix for each entry along with a new possibly shorter length, then combining all of the organism sequence data together into the output MCP file. Because an MCP is common to all of the sequences, only one sequence file needs to accessed for each MCP file; however all of the additional data for the multiple organisms is carried forward for easy reporting of final results.

#### Trim Maximal Common Prefixes to Produce Final Report

If our focus was purely on comparing two organisms then the algorithm to compute MCP's can retain just the longest sequence found. However with multiple organisms, when intersecting two MCP files, resultant MCP's may be shorter or substrings of the those in each individual file. Therefore, not only is the longest MCP stored in the file but also all of its suffix sequences down to the minimum length. If the intersection of two MCP files produces a match that is shorter, the suffix sequences maintained in sorted order allows the intersection process to be performed efficiently. At the end of the workflow, when all of the organisms have been intersected together, the shorter suffix sequences are no longer required. We provide the program, **trim_mcp**, which given an MCP file will produce an output MCP file with all shorter suffix sequences removed, leaving only the longest MCP's corresponding to the longest ultraconserved sequences shared by all the organisms; it also sorts the MCP's by length so that the longest sequences are displayed first. The program, **print_mcp**, will print out all of the MCP's in a given file in either a compact summary form, in FASTA format conducive for input into other programs, or in comma-delimited format for easy input into a spreadsheet program.

### Testing

We ran our algorithm and workflow on a number of example biological case studies to characterize the scalability and efficiency at which multiple organism whole genome comparative analysis can be performed. We describe some of the current algorithmic techniques and tools available, and compare our algorithm against three tools: BLAST, MUMmer, and Vmatch. Our algorithm outperforms or is similar to them in computation time and memory usage while providing additional benefits for multiple organism analyses not provided by any of the tools.

#### Ultraconserved sequences for 17 vertebrate genomes

We used our algorithm to reproduce the results of Bejerano *et al.*[6] by finding the 100% conserved nucleotide sequences between human, mouse, and rat. Our algorithm successfully found the 481 segments longer than 200 bp and 5524 segments longer than 100 bp in one day of computation on a modest computer cluster (8 machines).

Since the report by Bejerano et al., additional vertebrate genomes have been sequenced and assembled; currently there are 17 vertebrate genomes available (see Table 1). We used our algorithm to find the 100% conserved nucleotide sequences common to all of the genomes. The programs took about a week to run on our computer cluster producing 123 sequences longer than 40 bps with the longest being 104 bps mapping to the 40S ribosomal protein S6 (XR 018435).

**Table 1:** Vertebrate Genomes

Vertebrate Genomes		Size
Human	Mar. 2006 (hg18)	3000 Mbp
Chimp	Mar. 2006 (panTro2)	3100 Mbp
Rhesus	Jan. 2006 (rheMac2)	2800 Mbp
Chicken	May 2006 (galGal3)	1200 Mbp
Mouse	Mar. 2006 (mm8)	2500 Mbp
Rat	Nov. 2004 (rn4)	2800 Mbp
Cow	May 2005 (bosTau2)	3000 Mbp
Dog	May 2005 (canFam2)	2400 Mbp
Armadillo	May 2005 (dasNov1)	3000 Mbp
Elephant	May 2005 (loxAfr1)	3000 Mbp
Opossum	Jan. 2006 (monDom4)	3400 Mbp
Fugu	Aug. 2002 (fr1)	350 Mbp
Rabbit	May 2005 (oryCun1)	3500 Mbp
Zebrafish	Mar. 2006 (danRer4)	1700 Mbp
Tetraodon	Feb. 2004 (tetNig1)	380 Mbp
X. tropicalis	Aug. 2005 (xenTro2)	1700 Mbp
Tenrec	July 2005 (echTel1)	3800 Mbp

All vertebrate genomes with the given assembly data where downloaded from the UCSC Genome Bioinformatics Site [33]. Sizes from NCBI Genome Project web pages or approximated from downloaded assembly data.

#### Ultraconserved sequences within the human body louse and between three insect species

The human body louse, *Pediculus humanus humanus*, is an insect with a recently assembled genome of size slightly larger than 100 Mbp. As part of initial analysis of the genome, we ran our algorithm for the genome against itself and pairwise against three other insect genomes including *Drosophila melanogaster*, *Anopheles gambiae*, and *Apis mellifera*. We found 920,000 ultraconserved sequences that occur in multiple places within the louse genome; 85% of these correspond to repeat and low complexity regions which were not masked in the genome. We separated out the repeat sequences and separated out those sequences which overlapped the initial gene build for the genome, leaving 85,722 ultraconserved sequences longer than 40 bps. The longest sequence is 2646 bps with thousands of sequences that are longer than 300 bps. Work is continuing to also separate transposable elements from the list to get an accurate identification of non-coding ultraconserved elements in the body louse genome.

Pairwise comparative analysis with the three other insects produced 3431 total ultraconserved sequences between 40 and 100 bps in length. After separating sequences that overlapped both with the louse gene build and the other insect's genes, we are left with 1193 sequences. The total analysis for comparing the body louse against itself and against the three insects took just one day of computation time (not counting time to write a few BioPerl scripts for separating repeats and gene overlaps). The speed with which our algorithm performed clearly shows that such comparative analysis can be easily performed as part of the initial analysis of newly assembled genomes to identify potentially functional non-coding elements.

### Comparison to Other Methods

Comparison of genomic data is typically phrased in the terminology of aligning sequences whether this be local alignment versus global alignment, pairwise alignment for two sequences, or multiple alignment for multiple sequences. Furthermore, comparison is often in the context of protein coding sequences which implies that approximate computation techniques are required to find matches in the face of nucleotide mutations and degeneracy in the genetic code. Comparison techniques vary with tools like BLAST [16], BLASTZ [17], BLAT [18], and PatternHunter [19] allowing for relatively short query sequences to be aligned against a sequence database containing one or many large sequences; the focus of these tools are concerned primarily with performing adequate approximate matching in reasonable computation time. They tend to ignore multiple organisms and whole genome comparison requires the genome to be chopped up into small query sequences. Whole genome alignment tools like WABA [20] and LAGAN [21] including those that support multiple alignments like Multi-LAGAN [21] and MAVID [22] adequately consider handling large genome data; however because of their approximate matching techniques, they are computationally expensive for the simpler task of finding ultraconserved sequences. For this reason, tools like MUMmer [23], REPuter [24] and its successor Vmatch [25] use data structures such as suffix trees and suffix arrays that allow for efficient computation of exact matches. Regardless not all of these tools (MUMmer, REPuter) handle sequences in a memory-efficient manner and none provides direct support for a multiple organism whole genome workflow.

#### BLAST

With careful selection of parameters, specifically by disabling gaps, setting a large mismatch penalty, and optionally turning off masking of low complexity regions, BLAST [16] can be used to find 100% matches between two sequences. We ran some tests to compare the results, efficiency, and ease of use of our algorithm with BLAST. All tests were run on an idle PowerMac Dual G5 2.7 Ghz with 6 GB memory. We used 40 as the lower length bound for sequences.

For the first test, we compared chromosome 1 of the *Arabadopsis thaliana *nuclear genome to its chloroplast. A BLAST database of chromosome 1 (~30 Mbp) was made and the chloroplast genome (~150 Kbp) was the query sequence. Both BLAST and our algorithm performed the comparison very quickly, under one minute. Our algorithm returned 30 sequences while BLAST returned 22 sequences; the discrepancy in the results involved the forward and reverse strand. Generally one expects double the number of results because an ultraconserved sequence on one strand will also have an ultraconserved sequence on the complement strand, so the 30 sequences found by algorithm are 15 sequences on one strand and 15 on the complement. BLAST actually found all 15 sequences in a mix of forward and reverse strand results, but for some reason only managed to find 7 of the 15 complement sequences. It is possible that the results for the two strands can be different, when the ultraconserved sequence occurs in multiple places, as the leading and trailing ends may provide a longer match in one place versus another, so searching for matches on just one strand is technically not accurate though it is a good approximation. This situation did not occur with this test.

For the second test, we compared chromosome 1 of the chicken genome (~196 Mbp) with chromosome 1 of the human genome (~250 Mbp). A BLAST database was made with the human chromosome and the chicken chromosome was the query sequence. BLAST ran for approximately two hours before crashing with an out of memory error, no results were reported. Our algorithm ran for 19 minutes and produced 227 sequences.

To resolve the memory issues with BLAST programs, the query sequence can be split into small segments, Woolfe *et al.*[10] used 1 Mb segments for comparing *Fugu rubripes *to human, which are individually aligned and the results combined. Difficulties with this approach include providing sufficient overlap of segments so sequences on the border are not missed and the large number of results to be combined. Regardless this approach is computationally very expensive, we estimate BLAST would require around 13 hours to compare chicken chromosome 1 with human chromosome 1, and numerous result files would need to be parsed and combined to produce the final results.

Handling multiple organisms with BLAST is not implicitly supported but could be handled in a similar tournament-style fashion as with our algorithm. The results from running BLAST for two organisms would be combined together and formated into a BLAST database; the results from two other organisms are then used as query sequences against that database. This process would be repeated for as many organisms as desired. This would require an additional program to be written to parse and process the BLAST output; an additional step not required by our algorithm.

#### MUMmer 3.0

MUMmer [23,26,27] is a set of programs for rapidly aligning genomes that is similar to our algorithm in capability. It constructs a suffix tree data structure [28] for a given reference sequence, then the tree is traversed for a given query sequence to produce maximally match; these matches correspond to ultraconserved elements. Suffix trees are similar to suffix arrays but slightly more efficient because they can be constructed in *O*(*n*) time of the sequence length versus *O*(*nlgn*) for suffix arrays due to the sorting process. MUMmer's disadvantage is that it requires the whole tree to be present in physical memory. MUMmer 3.0 with its efficient suffix tree implementation requires 15.43 bytes of memory for each base pair, so the largest human chromosome 2 at 237.6 Mbp requires 3908 MB of memory; the very edge of addressable memory for 32-bit processors. With the finalized sequencing of the human genome filling in the gaps, chromosome 1 is now the largest at 252 Mbp and is too large for MUMmer to fit in 4 GB of memory. Various projects [29-31] have explored the issues of organizing suffix trees on disk.

We performed similar tests with MUMmer as with BLAST, comparing chromosome 1 of *Arabadopsis thaliana *to its chloroplast. With the nuclear genome as the reference sequence, MUMmer took 71 seconds while with the chloroplast genome as the reference sequence, it took only 48 seconds. Our algorithm was also sensitive to which sequence was used for the reference sequence taking 125 seconds for the nuclear genome and 43 seconds for the chloroplast genome.

For comparing chromosome 1 of the chicken genome to chromosome 1 of the human genome, we tried each as the reference sequence to MUMmer but in both cases the program aborted with an out of memory error (32-bit PowerMac G5 with 6 GB memory) during the construction of the suffix tree. Therefore, we ran our tests on a 64-bit Sun Dual Opteron 2.1 Ghz with 16 GB memory. Execution speed for MUMmer and our algorithm were about equal, both taking 10 minutes to compare the two chromosomes irregardless of which chromosome was used as the reference sequence. From watching the programs using the UNIX **top **command, our algorithm used only 500 MB memory (sequence data) while MUMmer used 3.7 GB memory.

As expected, the results show that if sufficient memory is available then the suffix tree data structure is faster than the suffix array. For large genomes, the difference is less apparent and our algorithm provides comparable execution time with a much smaller memory footprint. The lower memory requirement allows us to run two instances of our algorithm on each cluster machine taking advantage of both available processors. This can be especially important in the future with newer processors having multiple execution cores.

Our algorithm requires that the sequence data first be processed into a suffix array file. For *Arabadopsis thaliana *this was immediate for the chloroplast genome at under one second and took 4.5 minutes for the nuclear genome. Chicken chromosome 1 took 12 minutes to build the suffix array and 13 minutes for human chromosome 1. We point out that the cost for generating the initial suffix array is amortized across all of the comparisons that use the array, as the generation is only performed once; furthermore, multiple suffix arrays can be generated in parallel using a computer cluster.

#### Vmatch

Vmatch [25] is full-featured suite of tools that subsumes REPuter [24] and has improved time and space complexity through the use of enhanced suffix arrays [32]. Vmatch offers a number of matching and post-processing options for finding equivalent string matches between sequences including maximal unique (as with MUMmer), tandem, supermaximal, and complete matches, while post-processing provides inverse output (substrings not covered by the matches), masking, and clustering. It operates similar to BLAST and other tools whereby a database (set of index files) is constructed from one sequence set then query sequences are matched against that database. Vmatch can also perform matching of a sequence against itself. Like our algorithm, Vmatch uses suffix arrays which have better memory efficiency than suffix trees.

Vmatch was efficient in the construction of its database files requiring just a second for the *Arabadopsis thaliana *chloroplast, 1 minute for chromosome 1 of *Arabadopsis thaliana*, 3 minutes for chicken chromosome 1 of the chicken genome, and 3 minutes for human chromosome 1. However, we note that Vmatch only indexes one strand of the sequence and does not appear to allow both strands to be indexed together in its database files; though the reverse strand can be indexed into its own database. Analysis of ultraconserved elements requires finding matches against the forward and reverse strand separately, as well as provide forward and reverse query sequences, then combining the result files together. In our tests, use of the reverse strand index file produced no results requiring us to construct a reverse complement sequence first then create a database file with it, so in subsequent analysis, we just performed a single computation on the forward strand.

Vmatch took 13 seconds to find the 10 ultraconserved sequences for the forward strands of the *Arabadopsis thaliana *chromosome 1 with its chloroplast. Vmatch also showed different running times depending upon which sequence was used as the query versus the database. For comparing chromosome 1 of the chicken genome to chromosome 1 of the human genome, Vmatch took slightly over 2 minutes with the human chromosome as the query sequence and almost 3.5 minutes with the chicken chromosome as the query sequence. After considering the strand combinations to get full results, Vmatch is faster than our algorithm. Vmatch used about 1.3 GB memory while processing the chicken and human sequences which is less than required by MUMmer but more than our algorithm at 500 MB.

Vmatch is more efficient than our algorithm for constructing the suffix array files and similar but faster in execution speed for finding ultraconserved sequences. One possible reason for the speed difference is our algorithm produces more output in anticipation of the next stage of the workflow. The primary disadvantage, which is seen with all the tools we reviewed, is the lack of explicit support for multiple organisms; output results are not provided in a format allowing for a simple workflow provided by our algorithm in Figure 2. Additional scripts are required to parse the output results, extract the relevant substrings from the sequence, generate a new set of database files for the next stage of the workflow, and with every stage maintain substring metadata so that final results can be correlated back to the original sequences. All tasks that are handled automatically by our algorithm.

## Conclusion

With more genomes becoming available at a faster rate, whole genome comparative analysis for multiple organisms is both feasible and desirable in our search for biological knowledge. We argue that bioinformatic programs should be forward thinking by assuming analysis on multiple (and possibly large) genomes, then consider both memory and computational complexity during algorithm design to maximize scalability. Our algorithm provides a case study for how a compromise design with a trade-off of disk space versus memory space allows for efficient computation while only requiring modest computer resources. The key advantages of our algorithm are:

• It produces output files that can be directly used as input for the next stage of the computational pipeline. This completely eliminates the need to write additional "glue" programs to parse output files into a format required for later stages of the pipeline.

• By focusing on a specialized task, computation can be orders of magnitude more efficient than more general algorithms, e.g. BLAST, making what use to be unreasonable analysis, quite possible. This also frees up more time to analyze the results instead of waiting for the results.

• The algorithm works for any number of organisms. By maintaining the result files from previous analysis, incorporating new organisms into the analysis is quickly and easily done without requiring all of the previous analysis to be repeated.

While our focus has been on finding the longest ultraconserved elements among multiple genomes, in the process we discovered that the genome in the suffix array data structure can be used for other tasks. One example is searching for class II transposable elements which have the structure of inverted repeat sequences flanking both ends of the transposase sequence. By performing the search for MCP's for an organism on itself then restricting the results to be within a certain distance apart (typical distances are specific for transposon type), the inverted repeats can be quickly found throughout the whole genome.

In future work, there are some enhancements we would like to make. If we relax the restriction of 100% identity then our algorithm tends to approach BLAST in capability by incorporating mismatches and gaps, where at some point it is better to just use BLAST. However, a simple relaxation like allowing for a user specified identity level will allow for more matches to be found. We also intend to explore additional comparative analyses that can take advantage of the genome in the suffix array data structure, thus providing a suite of comparative tools for use with multiple organisms.

## Availability and requirements

We have provided our algorithm in the BioCocoa library which is the de facto Objective-C bioinformatics framework instead of starting a new software package. The main functionality is provided in two Objective-C classes, BCSuffixArray and BCMCP, which supports operations on suffix arrays and MCP files respectively. By putting the functionality in general purpose classes, we hope this provides added utility as other programs can utilize those classes for different bioinformatic analyses. The command line tools described in this article directly utilize those classes and are provided as a BioCocoa application.

• Project name: BioCocoa

• Project home page: 

• Operating system(s): Mac OS X, GNU/Linux

• Programming language: Objective-C

• Other requirements: GNUstep for GNU/Linux systems

• License: Creative Commons Share-Alike Attribution Version 2.5

• Any restrictions to use by non-academics: none

## Authors' contributions

SC designed and implemented the algorithm. SC and NL discussed and performed the biological analyses. SC, NL and GM wrote the paper.
